# Supplementary material for: Multivariate Prognostic Model for Predicting the Outcome of Critically Ill Patients Using the Aromatic Metabolites Detected by Gas Chromatography-Mass Spectrometry
Source: Molecules. 2022 Jul 26;27(15):4784. doi: 10.3390/molecules27154784 (PMC9331661; doi:10.3390/molecules27154784)
Supplement: Supplementary file 1 [file molecules-27-04784-s001.zip › molecules-1822080-supplementary.pdf]

Supplementary Materials

Multivariate Prognostic Model for Predicting the Outcome of Critically Ill Patients Using the Aromatic Metabolites Detected by Gas Chromatography-Mass Spectrometry

Alisa K. Pautova [1,*], Andrey S. Samokhin [2], Natalia V. Beloborodova [1] and Alexander I. Revelsky [2]

[1] Negovsky Research Institute of General Reanimatology, Federal Research and Clinical Center of Intensive Care Medicine and Rehabilitology, 25-2 Petrovka Str., 107031 Moscow, Russia; nvbeloborodova@yandex.ru
[2] Chemistry Department, Lomonosov Moscow State University, GSP-1, Leninskie gory 1-3, 119991 Moscow, Russia; andrey.s.samokhin@gmail.com (A.S.S.), sorbent@yandex.ru (A.I.R.)
* Correspondence: alicepau@mail.ru; Tel.: +7-905-773-8981

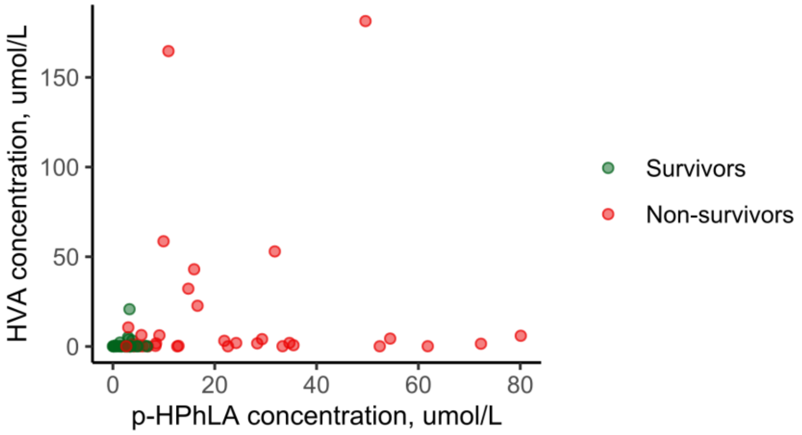

Figure S1. The concentration (μmol/L) of homovanillic (HVA) and 4-(3-hydroxyphenyl)lactic acids (*p*-HPhLA) in the blood serum of survivors (n = 44) and non-survivors (n = 35). Concentrations higher than 36 μmol/L are approximate because they were calculated by extrapolation.

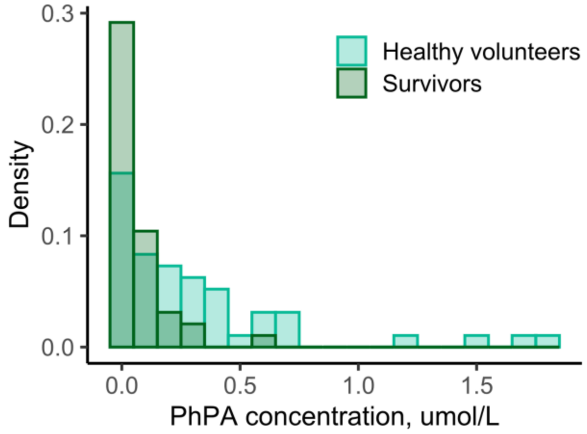

Figure S2. The distributions of phenylpropionic acid (PhPA) concentration, μmol/L, obtained for healthy volunteers (n = 52) and survivors (n = 44).

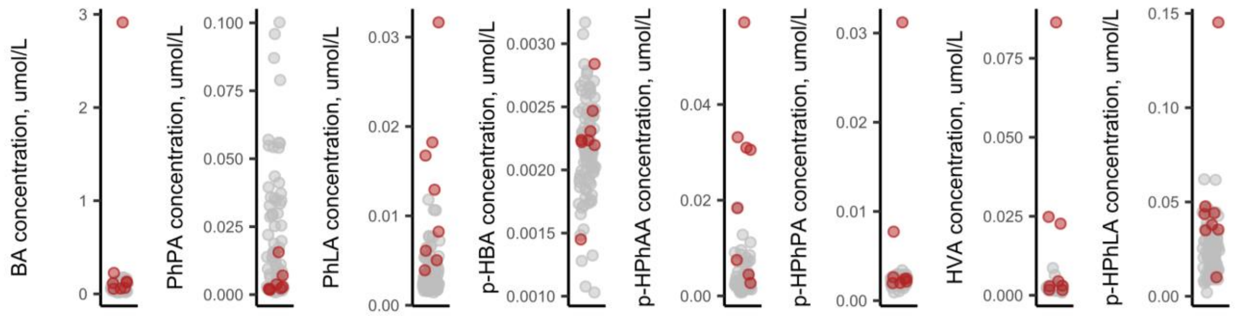

Figure S3: The concentration of all metabolites in the blood serum of survivors. Each person is represented by a single point. Eight samples (red points) are outliers. Points are scattered along the x-axis to improve perception in the case of overlapping. BA – benzoic acid; PhPA – 3-phenylpropionic acid; PhLA – 3-phenyllactic acid; *p*-HBA - 4-hydroxybenzoic acid, *p*-HPhAA – 2-(4-hydroxyphenyl)acetic acid; *p*-HPhPA – 4-(3-hydroxyphenyl)propionic acid; HVA – homovanillic acid; *p*-HPhLA – 4-(3-hydroxyphenyl)lactic acid.
